# Adult Seroprotection Gaps Against Diphtheria and Tetanus in Urban China: Repeated Cross-Sectional Serosurveillance in Pudong, Shanghai, 2017–2025

**DOI:** 10.3390/vaccines14070570

**Published:** 2026-06-29

**Authors:** Wanran Cheng, Juan Li, Tian Yang, Yu Bai, Pengfei Deng, Laibao Yang, Yihan Lu

**Affiliations:** 1 Shanghai Pudong New Area Center for Disease Control and Prevention (Shanghai Pudong New Area Health Supervision Institute), Shanghai 200136, China; m13914785746@163.com (W.C.); lijuan15180219@163.com (J.L.); tyang@pdcdc.sh.cn (T.Y.); 13364621799@163.com (Y.B.); masterdpf@163.com (P.D.); 2 Fudan University Pudong Institute of Preventive Medicine, Shanghai 200136, China; 3 Shanghai Institute of Infectious Disease and Biosecurity, Fudan University, Shanghai 200032, China; 4 Department of Epidemiology, Ministry of Education Key Laboratory of Public Health Safety, School of Public Health, Fudan University, Shanghai 200032, China

**Keywords:** diphtheria, tetanus, seroprotection, serosurveillance, adults, immunity gap

## Abstract

**Background**: Adult susceptibility to diphtheria and tetanus may increase as vaccine-induced immunity wanes, yet repeated population-based serosurveillance data in China are limited. **Methods**: We analyzed annual serosurveys conducted in Pudong New Area, Shanghai, China, from 2017 to 2025 among healthy adults aged 20–49 years. Diphtheria and tetanus IgG concentrations were measured by ELISA. Seroprotection was defined as antibody concentration ≥0.1 IU/mL. Antibody concentrations were further categorized as <0.01, 0.01–<0.1, 0.1–<1.0, and ≥1.0 IU/mL, and geometric mean concentrations (GMCs) were calculated. Multivariable logistic regression models were fitted to assess factors associated with non-protection, including survey year, age group, and household registration. Sensitivity analyses excluding the 2018 survey year were conducted. **Results**: A total of 2376 serum samples were included. Overall seroprotection was 21.46% for diphtheria and 13.80% for tetanus. The proportion protected against both antigens was 9.05%, while 73.78% showed concurrent non-protection against both antigens. The overall GMC was 0.032 IU/mL (95% CI: 0.030–0.034) for diphtheria and 0.018 IU/mL (95% CI: 0.017–0.019) for tetanus. Concentrations ≥1.0 IU/mL were uncommon for both antigens. Adults aged 40–49 years had higher odds of non-protection than those aged 20–29 years for diphtheria (OR: 2.43, 95% CI: 1.85–3.21) and tetanus (OR: 2.94, 95% CI: 2.11–4.13). Non-local residents also had higher odds of non-protection than local residents for diphtheria (OR: 1.55, 95% CI: 1.24–1.93) and tetanus (OR: 2.81, 95% CI: 2.15–3.69). Seroprotection varied across survey years, with a marked nadir in 2018. Sensitivity analyses excluding 2018 attenuated most year-specific associations, whereas age- and residence-related differences persisted. **Conclusions**: Healthy adults aged 20–49 years in Pudong showed low seroprotection and low GMCs against both diphtheria and tetanus, with a high proportion concurrently non-protected against both antigens. These findings highlight a persistent adult immunity gap and support further evaluation of adult booster strategies and enhanced serosurveillance.

## 1. Introduction

Diphtheria and tetanus are severe toxin-mediated infectious diseases caused by *Corynebacterium diphtheriae* and *Clostridium tetani*, respectively [1]. Although routine childhood immunization has substantially reduced the incidence of both diseases in many settings, vaccine-induced antitoxin levels may decline over time [2]. As a result, susceptible individuals can accumulate in older age groups despite sustained high childhood vaccination coverage.

Diphtheria has been well controlled in China through vaccination. For diphtheria, low circulation reduces observed disease, but susceptibility may become important if exposure risk increases through importation, population movement, or localised transmission in susceptible groups. In recent years, non-toxigenic C. diphtheria infections or carriage have been reported in several parts of China, including Dongyang, Guangzhou, and Zhuhai [3,4,5]. Recent respiratory diphtheria outbreaks in Germany [6] and Vietnam [7] have further highlighted that transmission can re-emerge among adolescents and adults when population immunity is insufficient. Unlike diphtheria, tetanus presents a unique challenge as it is not transmitted person-to-person and therefore cannot be controlled through herd immunity alone; prevention relies on maintaining adequate individual antitoxin levels across the life course [8]. According to WHO data, 85.25% of reported tetanus cases in 2024 were among adults (19,465/22,833) [9]. A retrospective study in the Anhui Province, China, reported that 97.85% of tetanus patients during 2013–2022 were aged over 18 years [10]. In China, cross-sectional studies have described age-related declines in seroprotection, but the population-level life-course trajectory of tetanus antibody waning remains insufficiently characterized [11,12]. Thus, low reported incidence does not necessarily indicate adequate adult protection, and serosurveillance provides an important complement to case-based surveillance for identifying adult immunity gaps.

In China, diphtheria-tetanus-pertussis (DTP) vaccination was introduced into the Expanded Program on Immunization (EPI) in 1978, and the immunization landscape transitioned from whole-cell pertussis vaccine (DTwP) to acellular pertussis vaccine (DTaP) starting in 2007, achieving coverage rates exceeding 95% [13]. The childhood schedule used for many years included four doses of diphtheria-tetanus-pertussis vaccine at 3, 4, and 5 months of age and at 18–24 months, followed by one dose of diphtheria-tetanus vaccine (DT) at 6 years of age [14]. From 1 January 2025, the national schedule was revised to five doses of diphtheria-tetanus-pertussis vaccine at 2, 4, 6, and 18 months and 6 years of age, replacing the previous 6-year diphtheria-tetanus booster [15]. However, the first generation of children immunized since the inception of the EPI is now approaching middle age. Despite the success of routine childhood schedules, adult protection is often compromised by waning antibody concentrations, heterogeneous booster recommendations, and limited documentation of adult booster uptake. Evidence from Australia [16], Singapore [17] and Thailand [18] has shown that a substantial proportion of adults lack adequate protection against diphtheria and tetanus, particularly between the ages of 30 and 50. Domestic studies in provinces such as Shaanxi [19] further confirm that even with high initial seropositivity, the depth of protection fluctuates significantly across different birth cohorts. However, most existing Chinese studies are either limited by small sample sizes or rely on single-year cross-sectional designs, which fail to distinguish long-term immunological trends from transient fluctuations caused by sampling bias or laboratory variability. These findings underscore a critical knowledge gap: high childhood coverage does not automatically translate into durable adult protection, particularly when decades have elapsed since the last scheduled booster.

Pudong New Area is a highly urbanised district of Shanghai with a large and mobile population, making it a relevant setting for assessing adult immunity in a metropolitan context. Population mobility may increase the complexity of local immunity patterns and has implications for both imported respiratory diphtheria risk and tetanus vulnerability in working-age adults. Therefore, we analysed repeated cross-sectional serosurveillance data collected from healthy adults aged 20–49 years in Pudong New Area, Shanghai, from 2017 to 2025. Using 0.1 IU/mL as the primary seroprotective threshold, we quantified year- and age-specific seroprotection against diphtheria and tetanus, characterised the proportion of adults with antibody concentrations ≥0.1 IU/mL for both antigens and those below this threshold for both antigens, and assessed the associations of age and survey year with non-protective antibody levels. Our findings aim to provide seroepidemiological evidence for understanding adult immunity gaps and to inform discussions on adult booster immunization strategies in urban China.

## 2. Materials and Methods

### 2.1. Study Design

A community-based serosurveillance was conducted in Pudong New Area, Shanghai, from 2017 to 2025. This study used a repeated cross-sectional design. Each year, 16 communities were selected from 48 communities in Pudong New Area using a random number table, and eligible adults aged 20–49 years were recruited from the selected communities. Selected communities could overlap across survey years; however, each annual survey was conducted as an independent cross-sectional sampling process. Within each survey year, participants were recruited according to the routine surveillance protocol. Available participant identifiers were checked, and no repeated individuals were identified across survey years. The study protocol was approved by the Ethics Committee of Shanghai Pudong New Area Center for Disease Control and Prevention (Shanghai Pudong New Area Health Supervision Institute) (Approval No.: PDCDCLL-20260402-012).

### 2.2. Sample Size

According to the “Shanghai Monitoring Plan for Population Immunity Levels and Vaccine Effectiveness” and the resident population structure in Pudong, participants were stratified into three adult age groups: 20–29 years, 30–39 years, and 40–49 years. The surveillance protocol targeted approximately 264 serum samples annually, with about 88 participants allocated to each of the three age groups. Due to field recruitment, the final number of eligible samples varied slightly across years and age groups. In total, 2376 serum samples from adults aged 20–49 years with valid diphtheria and tetanus antibody results were included in the final analysis.

### 2.3. Testing Method and Interpretation Criteria

Venous blood samples were collected from participants, and serum was separated and stored at −80 °C until testing. An enzyme-linked immunosorbent assay (ELISA; Virion/Serion GmbH, Würzburg, Germany) was used to measure anti-diphtheria toxoid IgG (REF: ESR130G) and anti-tetanus toxin IgG (REF: ESR108G) quantitatively. All assays were performed in accordance with the manufacturer’s operational instructions. The microtiter plates, negative controls, and kit-specific calibrators were supplied as components of the ELISA kits and were used according to the manufacturer’s instructions. Results were expressed in international units per millilitre (IU/mL).

According to the manufacturer’s instructions, IgG concentrations against diphtheria toxoid were classified into four categories: <0.01 IU/mL, indicating undetectable antibody or no protection; 0.01–<0.1 IU/mL, indicating insufficient protection; 0.1–<1.0 IU/mL, indicating basic or short-term protection; and ≥1.0 IU/mL, indicating long-term protection. IgG concentrations against tetanus toxoid were similarly classified into four categories: <0.01 IU/mL, indicating no detectable immunity; 0.01–<0.1 IU/mL, indicating that immune protection was not ensured; 0.1–<1.0 IU/mL, indicating adequate immune protection; and ≥1.0 IU/mL, indicating long-term protection. In seroepidemiological studies and immunization guidelines, antitoxin concentrations are commonly interpreted using two thresholds: ≥0.01 IU/mL indicates detectable antibody/seropositivity or low-level (partial) protection, whereas ≥0.1 IU/mL is widely used to define seroprotection (adequate protection) [20,21]. Because the public-health consequences of falling below the seroprotective threshold are more direct for clinical vulnerability and prevention planning, we adopted ≥0.1 IU/mL as the primary definition of protection in this study.

### 2.4. Statistical Analysis

Serum antibody concentrations were summarised by survey year, age group, sex and household registration. The proportions of participants with antibody concentrations below the protective threshold (<0.1 IU/mL) were calculated for diphtheria and tetanus. Antibody levels were presented as geometric mean concentrations (GMCs) with 95% confidence intervals (CIs). Differences in seroprotection indicators across participant characteristics were assessed using chi-square tests or Fisher’s exact tests, as appropriate.

Binary logistic regression models were fitted separately for diphtheria and tetanus, with non-protection as the outcome. The main models included age group, survey year and household registration as categorical covariates. Results are reported as odds ratio (OR) with 95% confidence interval (CI).

Sensitivity analyses excluding the 2018 survey year were conducted. All analyses were performed using R software (version 4.4.3), and two-sided *p* < 0.05 was considered statistically significant.

## 3. Results

### 3.1. Overall Seroprotection Status

Among 2376 adults aged 20–49 years surveyed during 2017–2025, 510 participants were seroprotected against diphtheria, corresponding to an overall seroprotection rate of 21.46%. For tetanus, 328 participants were seroprotected, with an overall seroprotection rate of 13.80%. Only 215 participants had antibody concentrations ≥0.1 IU/mL for both antigens (9.05%), whereas 1753 participants showed concurrent non-protection (73.78%). (Table 1) Seroprotection rates differed significantly by year, age group and household registration for both diphtheria and tetanus (both *p* < 0.05). Diphtheria seroprotection ranged from 9.85% in 2018 to 28.41% in 2019, while tetanus seroprotection ranged from 6.06% in 2018 to 23.11% in 2019. Diphtheria seroprotection decreased from 25.38% in the 20–29-year group and 24.49% in the 30–39-year group to 14.52% in the 40–49-year group. For tetanus, with seroprotection of 16.29%, 15.66%, and 9.47% in the 20–29, 30–39, and 40–49-year groups, respectively. The proportion protected against both antigens decreased from 12.63% and 11.11% in the 20–29 and 30–39-year groups to 3.41% in the 40–49-year group. No significant differences in seroprotection were observed by sex.

### 3.2. Seroprotection by Survey Year and Age Group

**Figure 1:**
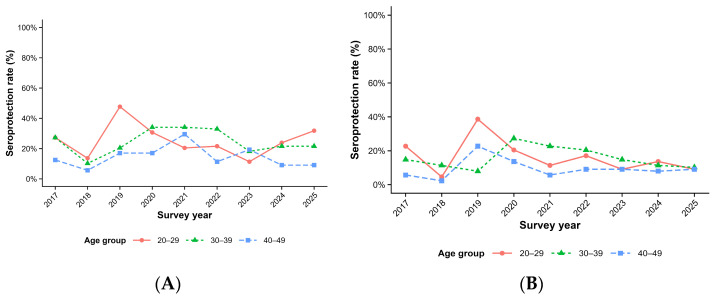
Annual seroprotection rates (≥0.1 IU/mL) against diphtheria and tetanus by age band among adults aged 20–49 years, Pudong, Shanghai, 2017–2025. (**A**) Diphtheria. (**B**) Tetanus.

Age-stratified annual seroprotection patterns are shown in Figure 1. Throughout the study period, seroprotection remained low in all age groups (20–29, 30–39, and 40–49 years),with the 40–49-year group generally showing the lower seroprotection than younger age groups, particularly for tetanus. A marked decline was observed in 2018, whereas most other year-to-year differences were smaller.

### 3.3. Antibody Concentration Distributions and GMCs

The distributions of diphtheria and tetanus IgG concentrations across predefined thresholds and GMCs are shown in Table 2. Overall, the GMC was 0.032 IU/mL (95% CI: 0.030–0.034) for diphtheria and 0.018 IU/mL (95% CI: 0.017–0.019) for tetanus. For diphtheria, most participants had antibody concentrations between 0.01 and <0.1 IU/mL [1459/2376, 61.41%], whereas 407 participants (17.13%) had concentrations <0.01 IU/mL and only 11 (0.46%) had concentrations ≥1.0 IU/mL. The lowest diphtheria GMC was observed in 2018 [0.008 IU/mL (95% CI: 0.007–0.011)], when 52.27% of participants had concentrations <0.01 IU/mL. Age-stratified analyses showed that diphtheria GMCs were lower among adults aged 40–49 years [0.026 IU/mL (95% CI: 0.024–0.029)] than among those aged 20–29 years [0.036 IU/mL (95% CI: 0.032–0.040)] and 30–39 years [0.034 IU/mL (95% CI: 0.030–0.037)]. Non-local residents also had a slightly lower diphtheria GMC than local residents [0.030 IU/mL (95% CI: 0.027–0.032) vs. 0.035 IU/mL (95% CI: 0.032–0.039)].

For tetanus, 854 participants (35.94%) had concentrations <0.01 IU/mL, 1194 (50.25%) had concentrations between 0.01 and <0.1 IU/mL, and only 28 (1.18%) had concentrations ≥1.0 IU/mL. The tetanus GMC was lowest in 2018 [0.005 IU/mL (95% CI: 0.004–0.007)], with 64.77% of participants below 0.01 IU/mL. Tetanus GMCs decreased across age groups, from 0.022 IU/mL (95% CI: 0.019–0.024) among adults aged 20–29 years to 0.015 IU/mL (95% CI: 0.014–0.017) among those aged 40–49 years. A less favorable distribution was also observed among non-local residents, who had a lower tetanus GMC than local residents [0.015 IU/mL (95% CI: 0.014–0.016) vs. 0.024 IU/mL (95% CI: 0.022–0.027)] and a higher proportion with concentrations <0.01 IU/mL [40.38% vs. 29.25%].

### 3.4. Factors Associated with Non-Protection

**Figure A1:**
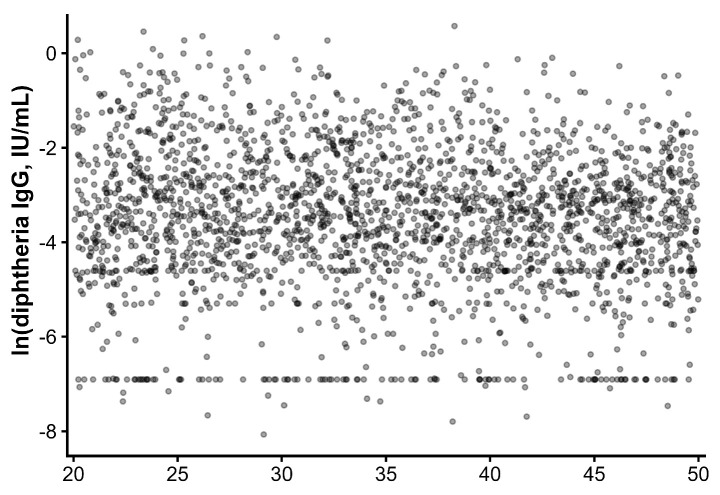
Scatterplot of log-transformed diphtheria antitoxin concentration versus age (20–49 years), Pudong, Shanghai, 2017–2025.

**Figure A2:**
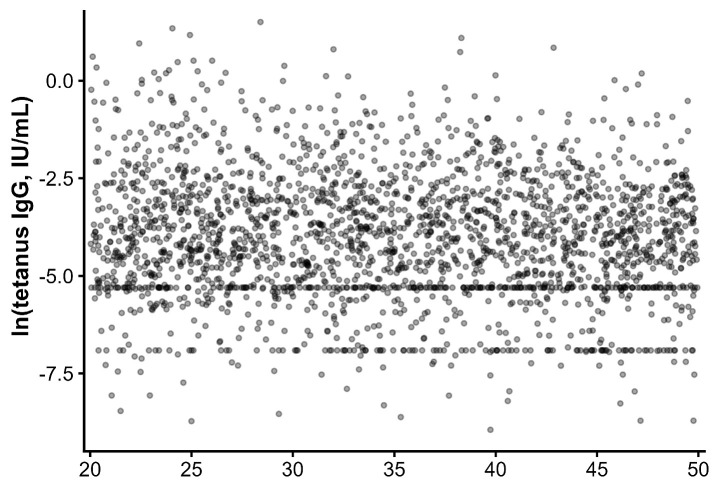
Scatterplot of log-transformed tetanus antitoxin concentration versus age (20–49 years), Pudong, Shanghai, 2017–2025.

In logistic regression models with non-protection as the outcome, age group and household registration was associated with non-protection (Table 3). Compared with adults aged 20–29 years, those aged 40–49 years had significantly higher odds of non-protection for both diphtheria (OR: 2.43, 95% CI: 1.85–3.21) and tetanus (OR: 2.94, 95% CI: 2.11–4.13). The 30–39 year group did not differ significantly from the 20–29-year group for either diphtheria (OR: 1.11, 95% CI: 0.87–1.41) or tetanus (OR: 1.26, 95% CI: 0.95–1.67). Compared with 2017, the 2018 survey was associated with higher odds of non-protection against both diphtheria and tetanus. For tetanus, lower odds of non-protection were observed in 2019 (OR: 0.49, 95% CI: 0.31–0.77) and 2020 (OR: 0.51, 95% CI: 0.31–0.81) compared with 2017, while most other survey years showed no statistically significant differences. Household registration was also associated with non-protection: non-local residents had higher odds of non-protection than local residents for both diphtheria (OR: 1.55, 95% CI: 1.24–1.93) and tetanus (OR: 2.81, 95% CI: 2.15–3.69). Sensitivity analyses excluding the 2018 survey year yielded similar estimates for the association between age group and non-protection (Table A1). The distribution of household registration varied across survey years (Table A2), and household registration was therefore included in multivariable models. Scatterplots of log-transformed antibody concentrations by age are provided in Figure A1 and Figure A2.

## 4. Discussion

In this repeated cross-sectional serosurveillance of adults aged 20–49 years in Pudong (2017–2025), we identified a substantial immunity gap for both diphtheria and tetanus. Using the predefined seroprotective threshold of 0.1 IU/mL, only 9.05% of participants achieved both antigens protection, while 73.78% demonstrated concurrent non-protection. This suggests that insufficient protection in adults is not limited to a single antigen, but reflects concurrent vulnerability to two toxoid-preventable infections that are commonly targeted together in routine vaccination schedules. The threshold-based distribution and GMC analyses further supported this finding: The overall GMC of diphtheria and tetanus IgG antibodies was 0.032 IU/mL and 0.018 IU/mL, respectively. Antibody concentrations were generally low for both antigens, high-level antibody concentrations were uncommon, and tetanus showed a lower overall GMC and a larger proportion of concentrations <0.01 IU/mL than diphtheria.

Our findings are consistent with previous Chinese seroepidemiological studies showing age-related declines in diphtheria and tetanus antibodies and insufficient protection among adults. An earlier study in Weifang found that the diphtheria antibody positivity rate dropped from 87.5% among people aged 18–24 to 74.4% among people aged 35–50 [22]. A serosurveillance study in Chongqing showed that the proportions of protective antibodies against diphtheria and tetanus decreased from 39.2% and 28.4% among adults aged 18–40 years to 29.0% and 8.1% among those aged 40–50 years, respectively [11]. When applying equivalent clinical stringency (≥0.1 IU/mL) and age-stratified metrics comparison, other economically developed regions of China exhibit similarly low adult seroprotection. In the Guangzhou serosurvey, age-stratified results showed that full seroprotection against diphtheria and tetanus among adults aged 21–30 years was 16.0% and 13.6%, respectively, and decreased further to 8.4% and 7.6% among those aged >40 years [23]. Similarly, a study in Zhejiang Province reported a tetanus seroprotection rate of 7.5% among adults aged 20–39 years [24]. Together, these findings suggest that the adult seroprotection gap observed in Pudong is not an isolated local phenomenon, but is broadly consistent with patterns reported in other Chinese settings.

Several factors may contribute to the low adult seroprotection observed in Pudong and other Chinese settings. In addition to methodological differences across studies, low adult seroprotection may partly reflect birth-cohort effects, historical variation in childhood immunization implementation, and population composition. Participants in this study were born approximately between 1968 and 2005, a period spanning the introduction and early expansion of China’s EPI in 1978 [25]. Adults in the older age groups were born before or around the early implementation period of the EPI, when standardized delivery of childhood DTP vaccination and immunization service capacity were still being established [26]. Therefore, some individuals in these birth cohorts may have received incomplete or delayed primary immunization. The local context of Pudong may also have contributed to heterogeneity in childhood immunization histories. Before the large-scale development and opening-up of Pudong in the 1990s, parts of the area were more rural or suburban than the established urban core of Shanghai [27]. In addition, rapid urbanization and substantial population mobility in subsequent decades may have resulted in a study population with diverse places of birth, childhood residence, and vaccination histories. These factors may partly explain why adult seroprotection in our cohort was lower than that reported in some other Chinese studies.

International evidence indicates that adult immunity gaps are common. Surveys from other countries varied widely by country and were generally worse for diphtheria than tetanus, with clear age effects [28]. Age-related declines were reported in Greek data, where seroprotection (≥0.1 IU/mL) was 31.5% for diphtheria and 59.50% for tetanus [29]. In Thailand, using the same ≥0.1 IU/mL threshold, tetanus seroprotection was substantially higher than diphtheria seroprotection (95.10% vs. 65.40%), with the lowest diphtheria seroprotection observed among adults aged 31–40 years [18]. This differs from our findings, in which tetanus seroprotection was lower than diphtheria seroprotection. Therefore, diphtheria and tetanus antibody dynamics may differ and may not be fully synchronous across populations.

Mechanistically, although diphtheria and tetanus toxoids are commonly administered together in childhood immunization schedules, co-administration does not imply identical long-term antibody persistence. Antibody levels against the two antigens may wane at different rates, and limited systematic booster opportunities may further contribute to concentrations below the protective threshold for both antigens. These processes could result in discordant seroprotection within the same adult population, even when the two antigens were commonly delivered together. A booster study demonstrated that although tetanus-diphtheria vaccination induced strong short-term protection against both antigens, long-term persistence differed: tetanus protection was maintained in all participants 5 years after booster vaccination, whereas 24% of young adults and 54% of elderly adults were again unprotected against diphtheria, indicating less durable immunity for diphtheria [30]. In addition, several studies suggest that long-term persistence is often less favorable for diphtheria than for tetanus, even after adult booster vaccination, which can further widen vulnerability to diphtheria as age increases [30,31]. A quantitative synthesis showed that time to fall below 0.1 IU/mL for diphtheria strongly depends on the cumulative number of doses received across the life course, supporting progressive waning when booster completion is suboptimal [32].

These differences should be considered because direct cross-country comparisons are affected by variation in sampling methods, population composition, laboratory assays, and the thresholds used to define protection. Policy context is also relevant: countries with structured adult booster schedules and stronger adult immunization implementation may maintain better long-term protection. Many high-income countries including the United States [33], Canada [34] and Finland [35], explicitly recommend periodic adult boosters for tetanus- and diphtheria-containing vaccines, often every 10 years starting at age 19 [36]. In addition, reimbursement policies and easier access to adult vaccination services may contribute to higher long-term protection in some countries. Belgium [37] and Germany [38] provide certain adult vaccinations including those against tetanus and diphtheria free of charge. In our setting, adults may receive tetanus-containing vaccines in the context of wound management or injury-related prophylaxis [39]; however, such opportunities are event-driven and selective rather than part of a routine population-wide adult booster schedule. Therefore, injury-related prophylaxis may boost immunity in some individuals but may not be sufficient to maintain high tetanus antibody levels across the entire adult population covered by this serosurvey.

The particularly low seroprotection observed in 2018 for both diphtheria and tetanus may reflect a combination of population-related and sampling factors rather than a true abrupt decline in population immunity. Because each annual serosurvey was conducted as an independent cross-sectional sampling process, differences in sampled communities and participant composition across years may have introduced variability in observed seroprotection levels. Household registration is particularly relevant in this context. The distribution of local and non-local residents varied across survey years, and non-local residence was associated with higher odds of non-protection in the multivariable models. Therefore, variation in household registration distribution may have partly contributed to the unusually low estimates observed in 2018, although it is unlikely to fully explain the year-specific nadir. This interpretation is also consistent with the high population mobility and demographic heterogeneity in Pudong. Non-local residents may differ from local residents in early-life residence, childhood vaccination history, duration of residence, and access to immunization services across the life course, all of which could influence measured antibody levels. In addition, sampling variability cannot be excluded, especially given the finite annual sample size in each independent survey.

Although reported diphtheria and tetanus incidence remains low in China, the public health relevance of low seroprotection should not be underestimated. For diphtheria, low incidence does not eliminate the risk of re-emergence when susceptible individuals, population movement, and exposure opportunities overlap. Recent international experience has shown that toxigenic *Corynebacterium* diphtheriae can cause outbreaks in low-incidence settings, including an outbreak reported in Germany among newly arrived migrants [40]. For tetanus, low incidence does not imply population protection because herd immunity does not operate. Surveillance in England during 2001–2014 reported an overall case-fatality rate of 11.0%, with all tetanus-associated deaths occurring in adults older than 45 years and none fully immunised [41]. Therefore, the low seroprotection and low GMCs observed in this study should be interpreted as a signal of adult serological susceptibility rather than as direct evidence of imminent disease risk. These findings support continued serosurveillance and further evaluation of adult booster strategies.

This study has limitations. First, individual vaccination histories were unavailable; thus, we could not disentangle incomplete primary immunization, limited booster uptake in adulthood, and immunological waning as the relative contributors to low antitoxin levels. Second, although participants were recruited through a structured community-based surveillance programme covering multiple communities in Pudong, the sample was not designed to be fully representative of all adults in Pudong or the broader Shanghai population. Given Pudong’s high population mobility and socioeconomic heterogeneity, the observed seroprotection profile may partly reflect local population composition, migration history, and surveillance-site characteristics. Therefore, the findings should not be directly generalized to all adults in Shanghai or to other urban areas in China. Third, Because communities and participants were sampled independently in each survey year, year-to-year variation may partly reflect differences in sampled communities and participant composition. In addition, this study assessed serological susceptibility based on IgG concentrations, and seroprotection thresholds should be interpreted as surrogate markers of immunity rather than direct measures of vaccine effectiveness, clinical protection, or disease risk. Despite these limitations, the consistently low seroprotection levels and the very low proportion with antibody concentrations ≥0.1 IU/mL for both antigens indicate a substantial adult immunity gap in this surveillance population. These findings support further evaluation of adult booster strategies and strengthening of serosurveillance approaches to better characterise and address gaps in diphtheria and tetanus immunity across the adult life course.

## 5. Conclusions

Adults aged 20–49 years in Pudong showed low seroprotection against both diphtheria and tetanus, with a high proportion having antibody concentrations below the protective threshold for both antigens and an age-related increase in non-protection. These findings indicate a persistent adult immunity gap. Strengthening life-course immunization through improved booster needs, better documentation of adult vaccination history, and continued serosurveillance may help reduce susceptibility in adult-age populations.

## Figures and Tables

**Table 1:** Seroprotection against diphtheria and tetanus among adults aged 20–49 years in Pudong, Shanghai, 2017–2025.

Character	Diphtheria		Tetanus		Concurrent			
	*n*	Dip Protect (%)	*n*	Tet Protect (%)	*n*	Protected Against Both Antigens (%)	*n*	Non-Protected Against Both Antigens (%)
Year								
2017	59	22.35 (17.74–27.75)	38	14.39 (10.67–19.14)	26	9.85 (6.81–14.04)	193	73.11 (67.45–78.10)
2018	26	9.85 (6.81–14.04)	16	6.06 (3.76–9.62)	9	3.41 (1.80–6.35)	231	87.50 (82.97–90.96)
2019	75	28.41 (23.31–34.13)	61	23.11 (18.43–28.55)	43	16.29 (12.32–21.22)	171	64.77 (58.84–70.29)
2020	72	27.27 (22.26–32.94)	54	20.45 (16.03–25.73)	37	14.02 (10.34–18.72)	175	66.29 (60.39–71.72)
2021	74	28.03 (22.96–33.73)	35	13.26 (9.69–17.88)	25	9.47 (6.50–13.61)	180	68.18 (62.34–73.51)
2022	58	21.97 (17.40–27.35)	41	15.53 (11.66–20.39)	34	12.88 (9.36–17.46)	199	75.38 (69.84–80.19)
2023	43	16.29 (12.32–21.22)	29	10.98 (7.76–15.33)	12	4.55 (2.62–7.78)	204	77.27 (71.85–81.92)
2024	48	18.18 (14.00–23.28)	29	10.98 (7.76–15.33)	18	6.82 (4.36–10.52)	205	77.65 (72.25–82.26)
2025	55	20.83 (16.37–26.13)	25	9.47 (6.50–13.61)	11	4.17 (2.34–7.31)	195	73.86 (68.25–78.79)
$\chi^{2}$	46.80		50.82		55.61		52.81	
*p*	<0.01		<0.01		<0.01		<0.01	
Age Group								
20–29	201	25.38 (22.47–28.52)	129	16.29 (13.88–19.02)	100	12.63 (10.49–15.12)	562	70.96 (67.70–74.01)
30–39	194	24.49 (21.63–27.61)	124	15.66 (13.29–18.35)	88	11.11 (9.11–13.49)	562	70.96 (67.70–74.01)
40–49	115	14.52 (12.24–17.15)	75	9.47 (7.62–11.71)	27	3.41 (2.35–4.91)	629	79.42 (76.46–82.09)
$\chi^{2}$	34.20		18.91		47.01		19.51	
*p*	<0.01		<0.01		<0.01		<0.01	
Sex								
Male	227	21.37 (19.01–23.94)	146	13.75 (11.81–15.95)	95	8.95 (7.37–10.81)	784	73.82 (71.10–76.38)
Female	283	21.54 (19.40–23.84)	182	13.85 (12.09–15.82)	120	9.13 (7.69–10.81)	969	73.74 (71.30–76.05)
$\chi^{2}$	0.92		0.01		0.02		0.01	
*p*	0.92		0.94		0.87		0.97	
Household Registration								
Local	225	23.76 (21.16–26.57)	180	19.01 (16.64–21.63)	115	12.14 (10.21–14.38)	657	69.38 (66.37–72.23)
Non-local	285	19.94 (17.95–22.10)	148	10.36 (8.88–12.04)	100	7.00 (5.79–8.44)	1096	76.70 (74.44–78.82)
$\chi^{2}$	4.92		35.82		18.32		15.76	
*p*	0.03		<0.01		<0.01		<0.01	
Total	510	21.46 (19.86–23.16)	328	13.80 (12.48–15.25)	215	9.05 (7.96–10.27)	1753	73.78 (71.97–75.51)

**Table 2:** Distribution of diphtheria and tetanus IgG concentrations and geometric mean concentrations among healthy adults aged 20–49 years in Pudong, Shanghai, 2017–2025.

**A. Diphtheria**						
**Group**	***n***	**GMC (95% CI), IU/mL**	**Proportion, *n* (%)**			
			**<0.01 IU/mL, *n* (%)**	**0.01–<0.1 IU/mL, *n* (%)**	**0.1–<1.0 IU/mL, *n* (%)**	**≥1.0 IU/mL, *n* (%)**
Survey year						
2017	264	0.031 (0.026–0.036)	27 (10.23)	178 (67.42)	58 (21.97)	1 (0.38)
2018	264	0.008 (0.007–0.011)	138 (52.27)	100 (37.88)	26 (9.85)	0 (0)
2019	264	0.053 (0.046–0.061)	6 (2.27)	183 (69.32)	73 (27.65)	2 (0.76)
2020	264	0.06 (0.052–0.068)	10 (3.79)	182 (68.94)	67 (25.38)	5 (1.89)
2021	264	0.035 (0.029–0.043)	48 (18.18)	142 (53.79)	72 (27.27)	2 (0.76)
2022	264	0.036 (0.031–0.042)	40 (15.15)	166 (62.88)	57 (21.59)	1 (0.38)
2023	264	0.027 (0.023–0.032)	60 (22.73)	161 (60.98)	43 (16.29)	0 (0)
2024	264	0.036 (0.032–0.042)	31 (11.74)	185 (70.08)	48 (18.18)	0 (0)
2025	264	0.032 (0.027–0.037)	47 (17.8)	162 (61.36)	55 (20.83)	0 (0)
Age group						
20–29	792	0.036 (0.032–0.04)	128 (16.16)	463 (58.46)	192 (24.24)	9 (1.14)
30–39	792	0.034 (0.03–0.037)	130 (16.41)	468 (59.09)	192 (24.24)	2 (0.25)
40–49	792	0.026 (0.024–0.029)	149 (18.81)	528 (66.67)	115 (14.52)	0 (0)
Household Registration						
Local	947	0.035 (0.032–0.039)	145 (15.31)	577 (60.93)	221 (23.34)	4 (0.42)
Non-local	1429	0.030 (0.027–0.032)	262 (18.33)	882 (61.72)	278 (19.45)	7 (0.49)
Total	2376	0.032 (0.030–0.034)	407 (17.13)	1459 (61.41)	499 (21)	11 (0.46)
**B. Tetanus**						
**Group**	***n* **	**GMC (95% CI), IU/mL**	**Proportion, *n* (%)**			
			**<0.01 IU/mL, *n* (%)**	**0.01–<0.1 IU/mL, *n* (%)**	**0.1–<1.0 IU/mL, *n* (%)**	**≥1.0 IU/mL, *n* (%)**
Survey year						
2017	264	0.024 (0.02–0.028)	65 (24.62)	161 (60.98)	35 (13.26)	3 (1.14)
2018	264	0.005 (0.004–0.007)	171 (64.77)	77 (29.17)	13 (4.92)	3 (1.14)
2019	264	0.042 (0.036–0.05)	31 (11.74)	172 (65.15)	54 (20.45)	7 (2.65)
2020	264	0.048 (0.042–0.055)	10 (3.79)	200 (75.76)	48 (18.18)	6 (2.27)
2021	264	0.025 (0.022–0.03)	55 (20.83)	174 (65.91)	33 (12.5)	2 (0.76)
2022	264	0.017 (0.014–0.021)	116 (43.94)	107 (40.53)	40 (15.15)	1 (0.38)
2023	264	0.013 (0.01–0.015)	127 (48.11)	108 (40.91)	26 (9.85)	3 (1.14)
2024	264	0.013 (0.011–0.016)	136 (51.52)	99 (37.5)	28 (10.61)	1 (0.38)
2025	264	0.012 (0.01–0.015)	143 (54.17)	96 (36.36)	23 (8.71)	2 (0.76)
Age group						
20–29	792	0.022 (0.019–0.024)	257 (32.45)	406 (51.26)	111 (14.02)	18 (2.27)
30–39	792	0.019 (0.017–0.021)	295 (37.25)	373 (47.1)	117 (14.77)	7 (0.88)
40–49	792	0.015 (0.014–0.017)	302 (38.13)	415 (52.4)	72 (9.09)	3 (0.38)
Household Registration						
Local	947	0.024 (0.022–0.027)	277 (29.25)	490 (51.74)	164 (17.32)	16 (1.69)
Non-local	1429	0.015 (0.014–0.016)	577 (40.38)	704 (49.27)	136 (9.52)	12 (0.84)
Total	2376	0.018 (0.017–0.019)	854 (35.94)	1194 (50.25)	300 (12.63)	28 (1.18)

**Table 3:** Adjusted odds ratios for non-protection against diphtheria and tetanus by age group, survey year, and Household Registration.

Character	Diphtheria				Tetanus			
	OR	95% CI	95% CI	*p*	OR	95% CI	95% CI	*p*
Year								
2017 (reference)	1.00				1.00			
2018	2.43	1.85	3.21	<0.01	2.12	1.15	4.05	<0.01
2019	0.68	0.46	1.02	0.06	0.49	0.31	0.77	<0.01
2020	0.70	0.47	1.05	0.08	0.51	0.31	0.81	<0.01
2021	0.69	0.46	1.03	0.07	0.95	0.57	1.58	0.85
2022	0.87	0.57	1.33	0.53	0.61	0.37	1.01	0.06
2023	1.30	0.83	2.04	0.26	0.95	0.56	1.64	0.86
2024	1.29	0.84	1.99	0.25	1.35	0.81	2.29	0.27
2025	0.97	0.63	1.48	0.88	1.18	0.68	2.07	0.56
Age Group								
20–29 (reference)	1.00				1.00			
30–39	1.11	0.87	1.41	0.37	1.26	0.95	1.67	0.12
40–49	2.43	1.85	3.21	<0.01	2.94	2.11	4.13	<0.01
Household Registration								
Local	1.00				1.00			
Non-local	1.55	1.24	1.93	<0.01	2.81	2.15	3.69	<0.01

## Data Availability

Restrictions apply to the availability of these data, which were collected from the Shanghai Immunization Information System. Data are available from the authors with permission from the Shanghai Pudong New Area Center for Disease Control and Prevention (Shanghai Pudong New Area Health Supervision Institute).

## Abbreviations

DTP	Diphtheria-Tetanus-Pertussis
EPI	Expanded Program on Immunization
DTwP	Diphtheria, Tetanus, and Whole-Cell Pertussis Vaccine
DTaP	Diphtheria, Tetanus, and Acellular Pertussis Vaccine

## Appendix A

### Appendix A.1

**Table A1:** Sensitivity analysis excluding the 2018 survey results for non-protection (<0.1 IU/mL) against diphtheria and tetanus.

Character	Diphtheria				Tetanus			
	OR	95% CI	95% CI	*p*	OR	95% CI	95% CI	*p*
Year								
2017(reference)	1.00				1.00			
2019	0.69	0.46	1.03	0.07	0.49	0.31	0.78	<0.01
2020	0.70	0.47	1.05	0.08	0.51	0.32	0.81	0.01
2021	0.70	0.47	1.04	0.08	0.96	0.58	1.60	0.88
2022	0.88	0.58	1.35	0.57	0.62	0.38	1.03	0.07
2023	1.30	0.83	2.04	0.25	0.95	0.56	1.64	0.87
2024	1.29	0.84	1.99	0.25	1.36	0.81	2.33	0.25
2025	0.97	0.63	1.49	0.89	1.19	0.69	2.08	0.54
Age Group								
20–29	1.00				1.00			
30–39	1.08	0.85	1.36	0.56	1.30	0.97	1.74	0.08
40–49	2.37	1.78	3.15	<0.01	2.91	2.08	4.11	<0.01
Household Registration								
Local	1.00				1.00			
Non-local	1.50	1.19	1.89	<0.01	2.69	2.04	3.57	<0.01

**Table A2:** Distribution of household registration by survey year among healthy adults aged 20–49 years in Pudong, Shanghai, 2017–2025.

Survey Year	N	Local	Non-Local	$\chi^{2}$	*p*
2017	264	152 (57.58)	112 (42.42)	153.46	<0.01
2018	264	79 (29.92)	185 (70.08)		
2019	264	122 (46.21)	142 (53.79)		
2020	264	107 (40.53)	157 (59.47)		
2021	264	124 (46.97)	140 (53.03)		
2022	264	62 (23.48)	202 (76.52)		
2023	264	67 (25.38)	197 (74.62)		
2024	264	153 (57.95)	111 (42.05)		
2025	264	81 (30.68)	183 (69.32)		
